# Early clinical outcomes of the trans-ocular threading technique for four-point flanged scleral fixation of intraocular lenses: a case series

**DOI:** 10.1186/s12886-026-05197-7

**Published:** 2026-08-07

**Authors:** Joo-Ye Park, Woong-Joo Whang, Jae-Jung Kim, Ji-Yun Song, So-Hyang Chung, Hyun-Seung Kim, Yong-Soo Byun

**Affiliations:** 1 Department of Ophthalmology, Seoul St. Mary’s Hospital, College of Medicine, The Catholic University of Korea, Seoul, South Korea; 2 Department of Ophthalmology, Yeouido St. Mary’s Hospital, College of Medicine, The Catholic University of Korea, Seoul, South Korea; 3 Department of Ophthalmology, Seoul St. Mary’s Hospital, 222 Banpo-daero, Seocho-gu, Seoul, 06591 South Korea

**Keywords:** Scleral fixation, Flanged technique, Intraocular lens, Aphakia, IOL dislocation

## Abstract

**Purpose:**

To evaluate the early clinical outcomes and surgical feasibility of a trans-ocular threading technique for four-point flanged scleral fixation of intraocular lenses (IOLs).

**Methods:**

This retrospective case series included 12 eyes of 12 patients who underwent four-point flanged scleral fixation using a trans-ocular threading technique with a preloaded four closed-loop haptic intraocular lens (Artis PE, Cristalens, France). A bent 25-mm long 30-gauge needle was introduced 2.5 mm posterior to the limbus and passed across the anterior chamber to a contralateral corneal incision. During IOL delivery, the needle was threaded through the haptic loops, allowing extraocular suture externalization without intraocular suture handling. A 6 − 0 polypropylene suture (Prolene, Ethicon, USA) was secured using a flanged technique with low-temperature cautery. The anterior chamber was maintained with viscoelastic without an infusion system. Clinical outcomes, including corrected distance visual acuity (CDVA), intraocular pressure (IOP), corneal endothelial cell density (ECD), IOL centration, and postoperative complications, were evaluated at a mean follow-up of 3.2 ± 1.1 months (range, 2–5 months).

**Results:**

At a mean follow-up of 3.2 months (range, 2–5 months), corrected distance visual acuity (CDVA) improved in 10 of 12 eyes (83.3%), with a mean improvement from 0.86 ± 0.72 to 0.38 ± 0.51 logMAR. After suture revision in one eye with early postoperative IOL subluxation due to insufficient suture tension, all IOLs were well-centered at the final follow-up. Mean preoperative and postoperative IOP did not differ significantly (14.9 ± 2.4 vs. 14.1 ± 2.8 mmHg; *P* = 0.43). Mean corneal endothelial cell density (ECD) was preserved at final follow-up (2205 ± 907 vs. 2146 ± 926 cells/mm²; *P* = 0.61). Postoperative IOL decentration assessed by anterior segment optical coherence tomography (AS-OCT) was 0.31 ± 0.13 mm (range, 0.04–0.46 mm), with all 12 eyes below the 0.5 mm threshold for clinical significance. One eye developed transient hyphema that resolved spontaneously within one week. No cases of cystoid macular edema, hypotony, or significant intraocular inflammation were observed.

**Conclusions:**

The trans-ocular threading technique for four-point flanged scleral fixation demonstrated favorable early clinical outcomes with minimal complications. This approach may reduce intraocular manipulation and maintain anterior chamber stability without the need for an infusion system.

**Supplementary Information:**

The online version contains supplementary material available at https://doi.org/10.1186/s12886-026-05197-7.

## Introduction

In recent years, several sutureless techniques have been developed as alternatives to conventional suture-based scleral fixation of intraocular lenses (IOLs) [1–3], aiming to reduce suture-related complications and improve long-term stability [2, 5]. Among these, the Yamane flanged intrascleral fixation technique, which employs a double-needle technique with two-point haptic fixation using a three-piece IOL, has gained widespread popularity owing to its sutureless design and favorable outcomes [4, 5]. However, the Yamane technique requires precise intraocular manipulation for haptic docking and carries a risk of IOL tilt or decentration due to its two-point fixation geometry [5, 10]. An alternative approach using polypropylene sutures with thermal flanges through closed-loop haptics was introduced by Canabrava et al., demonstrating excellent long-term IOL stability with a knotless design [3, 6]. 

Subsequent modifications, including four-point flanged fixation using four closed-loop haptics (Artis PE, Cristalens, France), have enabled improved IOL centration with enhanced resistance to tilt and implantation through small corneal incisions (2.2–2.4 mm) [7, 8]. Comparative studies have suggested that four-point fixation may provide more predictable refractive outcomes than the Yamane technique, with lower rates of IOL tilt attributed to the additional fixation points [9]. Nevertheless, intraocular suture manipulation through closed-loop haptics remains technically demanding, particularly in eyes with poor visualization, miotic or damaged pupils, or when an infusion system is unavailable.

Therefore, this study aimed to evaluate the early clinical outcomes and feasibility of a modified trans-ocular threading technique for four-point flanged scleral fixation. This technique minimizes intraocular suture handling by enabling extraocular suture engagement following trans-ocular needle passage, potentially improving surgical safety and anterior chamber stability.

## Materials and methods

This retrospective case series was approved by the Institutional Review Board of Seoul St. Mary’s Hospital (KC25RISI0753) and adhered to the tenets of the Declaration of Helsinki. Patients who underwent four-point flanged scleral fixation of IOL using the trans-ocular threading technique between December 2024 and May 2025 were included.

Surgical indications included IOL subluxation (partial visibility of the IOL optic within the pupillary space), IOL dislocation (complete displacement of the IOL optic from the pupillary space), and aphakia. Clinical data including corrected distance visual acuity (CDVA), intraocular pressure (IOP), corneal endothelial cell density (ECD) measured by specular microscopy, surgical indications, predisposing factors for IOL subluxation, and postoperative complications were reviewed. IOL centration was assessed both clinically at the slit lamp under pharmacological dilation and quantitatively using anterior segment optical coherence tomography (AS-OCT; ANTERION, Heidelberg Engineering, Heidelberg, Germany). The pupil center offset (x and y coordinates) provided by the AS-OCT software was used to calculate the total IOL decentration magnitude as √(x² + y²). A decentration of ≥ 0.5 mm was considered clinically significant.

A statistical comparison of preoperative and postoperative CDVA, IOP, and ECD was performed using the Wilcoxon signed-rank test. A P value < 0.05 was considered statistically significant.

### Surgical technique

All surgeries were performed by a single experienced anterior segment surgeon under retrobulbar anesthesia. The Artis PE (Cristalens, Lannion, France) intraocular lens was used in all cases. This is a hydrophobic acrylic, single-piece, foldable IOL with four closed-loop (eyelet) haptics, a 6.0-mm optic, and an overall diameter of 13.0 mm. It can be injected through a corneal incision as small as 2.2 mm and is designed to distribute fixation forces evenly across four scleral points, providing enhanced rotational stability and resistance to tilt compared with two-point fixation designs. The Carlevale IOL (FIL-SSF; SOLEKO, Pontecorvo, Italy), another option for sutureless scleral fixation via T-shaped haptics, was not used in this series; differences between the two platforms are discussed below.

In eyes with a dislocated IOL or associated vitreous prolapse, anterior vitrectomy was performed prior to IOL fixation via a limbal approach using a 23-gauge anterior vitrectomy probe (CENTURION Vision System, Alcon, Fort Worth, TX, USA).

**Fig. 1:**
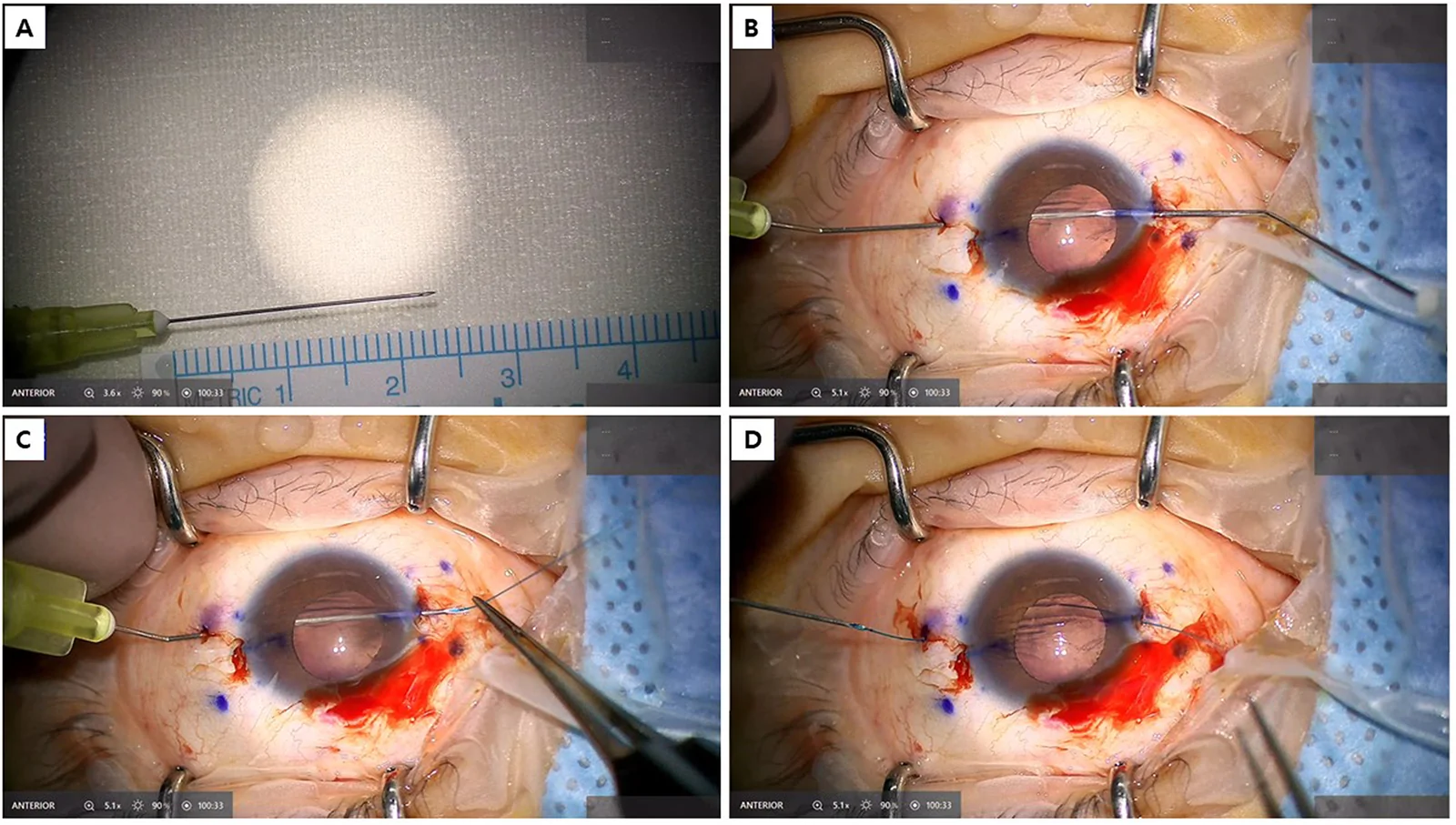
25-mm-long 30-gauge needle (**A**). Full trans-ocular passage of a needle was externalized through the opposite corneal incision (**B**). Extraocular suture engagement (**C**). The one end of suture was withdrawn with the needle through the original scleral entry (**D**)

The central horizontal meridian (0°–180°) was marked using a radial marker, and 1-mm clear limbal paracenteses were created at the nasal and temporal limbus bilaterally. Two scleral needle entry sites were marked 2.5 mm posterior to the limbus on each side to position the IOL near the ciliary sulcus plane while reducing the risk of iris–IOL contact [11]. The anterior chamber was filled with a dispersive viscoelastic (Sodium Hyaluronate 1.5%, Hyalu, Hanmi Pharm, Seoul, Korea) without the use of an infusion system. A bent 25-mm (1-inch) long 30-gauge needle (Sung-Shim Medical Co. Ltd., Daegu, Korea) was introduced at the nasal distal scleral entry site and advanced obliquely across the anterior chamber. A 27-gauge blunt cannula attached to the viscoelastic syringe was placed at the opposite temporal paracentesis to guide smooth externalization of the needle tip. Once externalized, the end of a 6 − 0 polypropylene suture (Prolene, Ethicon, Raritan, NJ, USA) was inserted into the lumen of the externalized needle, and the needle was withdrawn through the original scleral entry, delivering one suture end extraocularly (Fig. 1). To minimize sclerotomy size and reduce the risk of conjunctival complications, a 30-gauge needle and 6 − 0 polypropylene suture were utilized, ensuring adequate suture passage and secure thermal-cautery flange seating without unnecessary bulk.

**Fig. 2:**
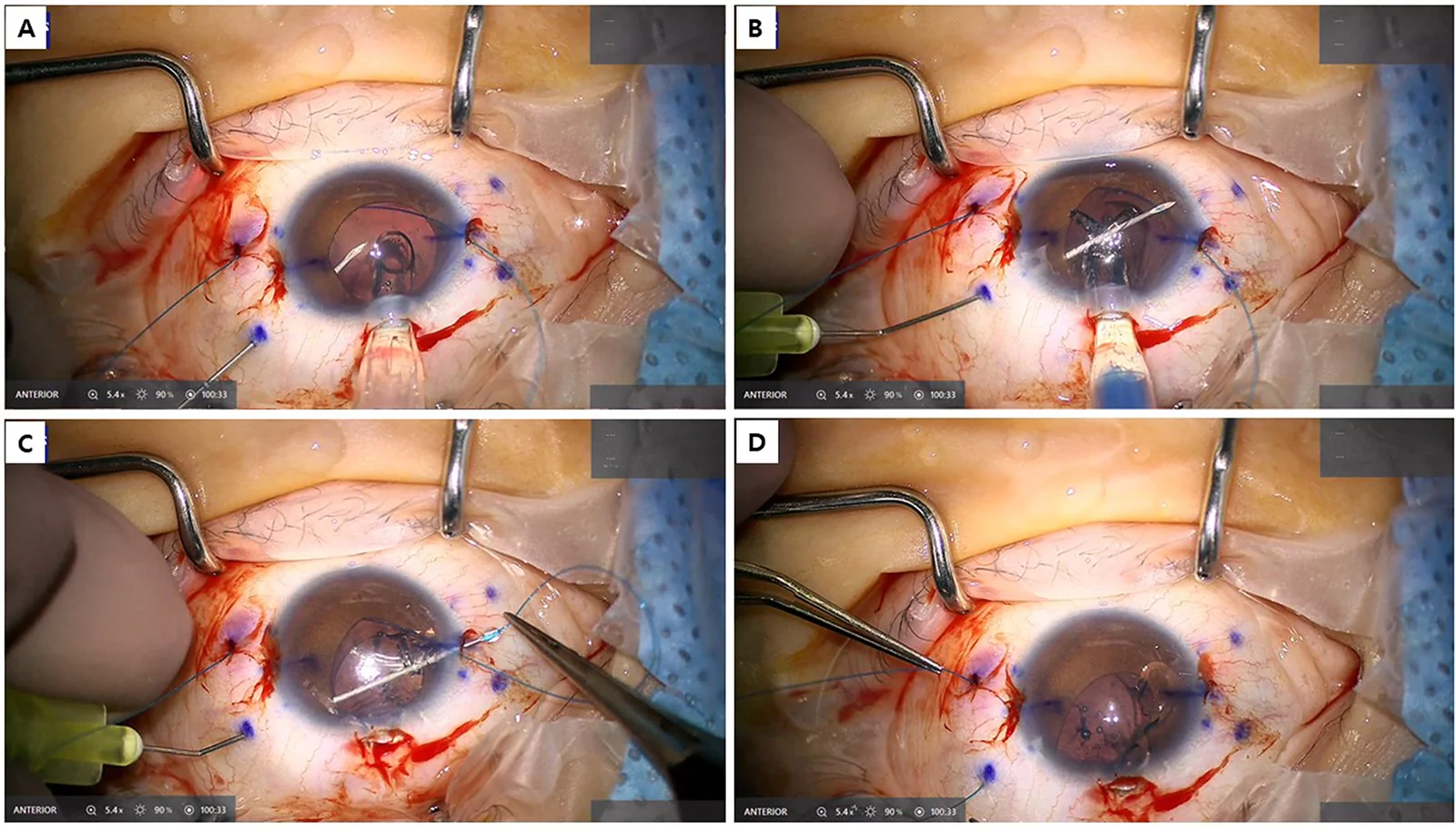
A 30-gauge long needle was inserted through the nasal proximal scleral entry site, while the cartridge preloaded with the 4-closed loop foldable IOL was engaged through the main incision (**A**). Intraocular threading of the leading haptic loop (**B**). After completing IOL loading in the anterior chamber and externalizing the needle, the other end of suture was engaged (**C**). After withdrawing the suture through scleral entry site, IOL was rotated and positioned (**D**)

The second trans-ocular needle pass was performed during IOL delivery to thread the leading haptic loops with the opposite end of the same suture. Through a 2.2–2.4 mm superior limbal incision, the IOL inserter was introduced. As the leading haptic loops emerged, a 30-gauge needle introduced through the nasal proximal scleral entry was passed through both loops and externalized. The opposite suture end was withdrawn nasally, completing fixation of the leading haptic, and sutures were tensioned to center the IOL (Fig. 2).

**Fig. 3:**
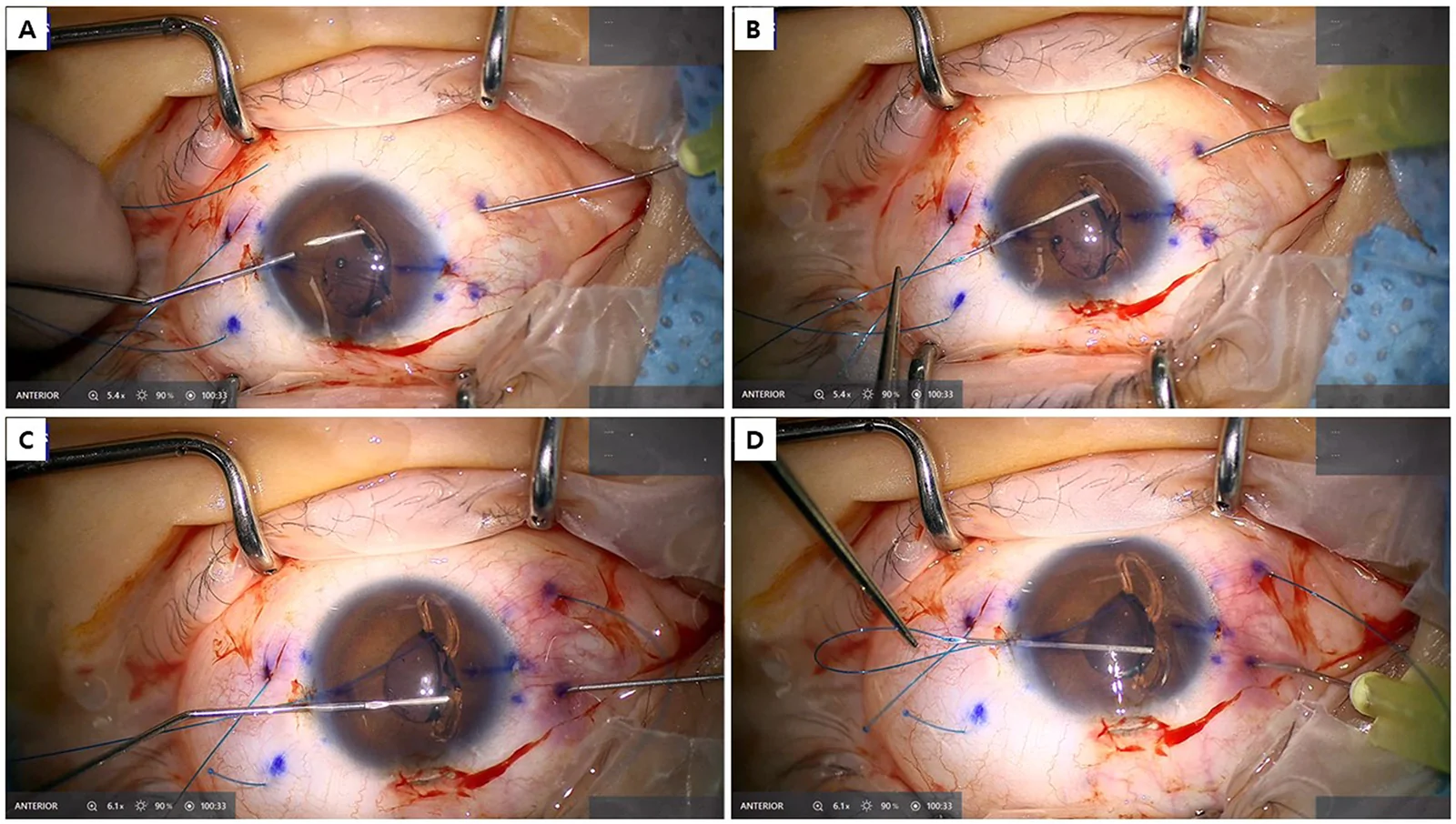
On the other side, the same procedure was repeated. Intraocular threading through one loop of the following haptic and externalization of a long needle through the opposite corneal incision (**A**). Suture was engaged and was withdrawn through the scleral entry with a long needle (**B**). Intraocular threading through the other loop of the following haptic and externalization of the needle (**C**). The other end of suture was engaged in the lumen of the needle, and delivered through the opposite scleral entry site (**D**)

The same process was repeated for the trailing haptic using the temporal scleral entry sites, completing suture passage through both trailing loops (Fig. 3).

**Fig. 4:**
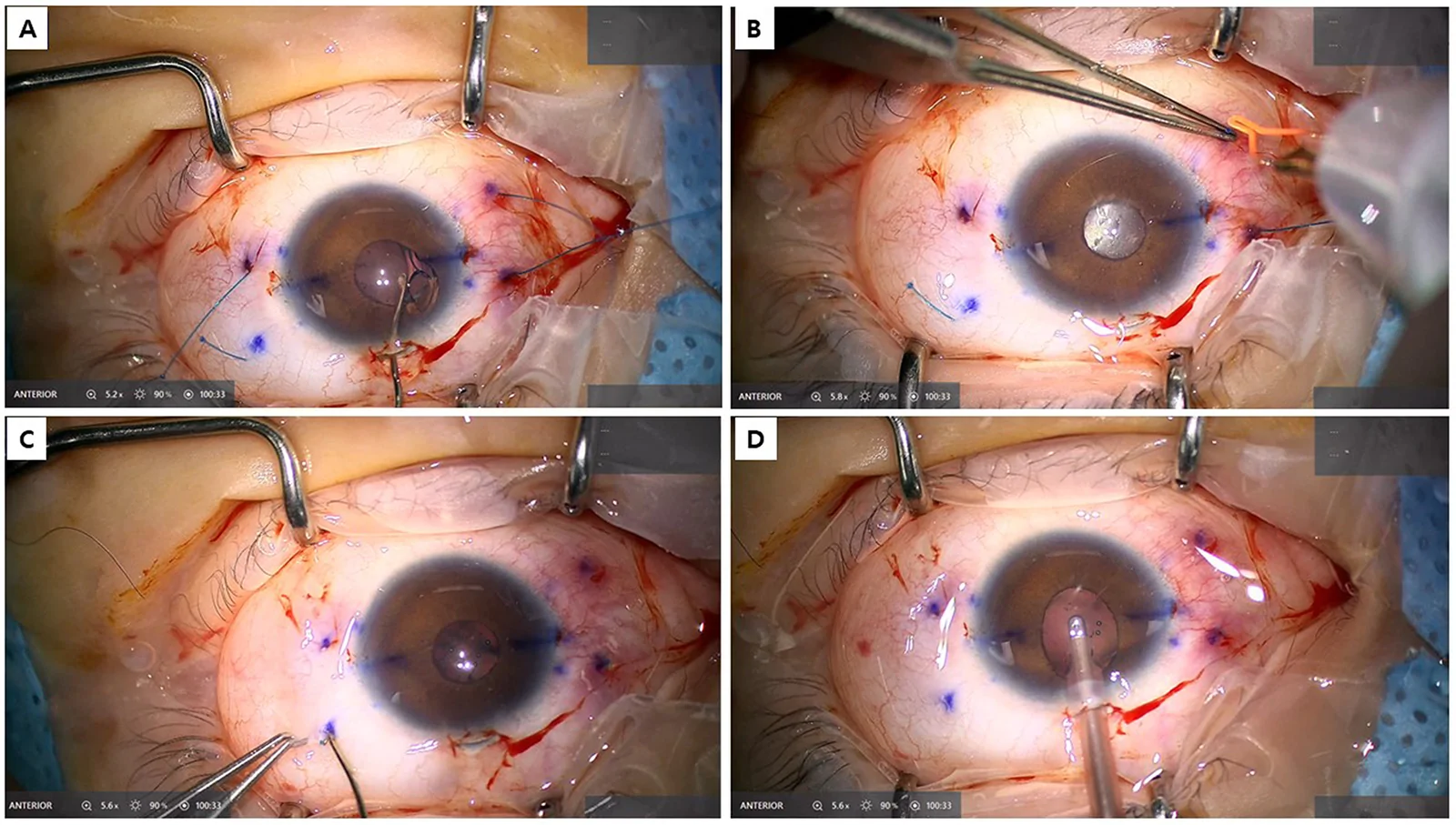
IOL centration (**A**). The IOL is positioned properly at the center with appropriate tension and four suture ends are flanged (**B**). The flanges are buried at the scleral sites using an instrument (**C**). The anterior chamber is inspected for vitreous prolapse, residual OVDs are removed, and the surgery is completed (**D**)

Four-point fixation was achieved. The IOL was positioned behind the iris, all sutures tensioned for stable centration, flanged with low-temperature cautery, and embedded into the sclera. Residual viscoelastic was removed and wound integrity confirmed (Fig. 4).

This infusion-free technique achieves four-point scleral fixation without intraocular suture manipulation, maintaining globe stability throughout and remaining feasible even in eyes with compromised pupils, iris defects, or reduced visualization.

## Results

A total of 12 patients (7 males, 5 females) with a mean age of 58.4 ± 17.8 years (range, 19–84 years) underwent four-point flanged scleral fixation using the trans-ocular threading technique. The mean follow-up duration was 3.2 ± 1.1 months (range, 2–5 months). The most common indication was IOL dislocation (*n* = 8, 66.7%), followed by aphakia (*n* = 3, 25.0%), and zonulysis (*n* = 1, 8.3%).

Four patients (33.3%) had concurrent corneal comorbidities that compromised surgical visualization, including pseudophakic bullous keratopathy (*n* = 2), granular corneal dystrophy (*n* = 1), and corneal opacity (*n* = 1). CDVA improved in 10 of 12 eyes (83.3%), with a mean improvement from 0.86 ± 0.72 to 0.38 ± 0.51 logMAR. Postoperatively, 8 eyes (66.7%) achieved a CDVA of 0.30 logMAR or better, and 4 eyes (33.3%) achieved 0.10 logMAR or better. Two eyes showed no visual improvement: one due to pre-existing granular corneal dystrophy with amblyopia (Case 1), and one with preoperative CDVA of 0.10 logMAR that remained unchanged postoperatively (Case 8), attributed to pre-existing macular pathology.

Intraocular pressure remained stable throughout the follow-up period, with mean preoperative and postoperative IOP values of 14.9 ± 2.4 and 14.1 ± 2.8 mmHg, respectively (*P* = 0.43, Wilcoxon signed-rank test). No eye developed sustained intraocular pressure elevation or hypotony during the observation period. Corneal ECD was preserved at final follow-up, with mean values of 2205 ± 907 cells/mm² preoperatively and 2146 ± 926 cells/mm² postoperatively (*P* = 0.61; *n* = 11; Case 12 excluded due to corneal pathology precluding specular microscopy).

IOL centration was evaluated quantitatively using AS-OCT in all 12 eyes. Mean postoperative IOL decentration, calculated from the pupil center offset, was 0.31 ± 0.13 mm (range, 0.04–0.46 mm). All 12 eyes were below the 0.5 mm threshold considered clinically significant for IOL decentration [9], consistent with well-centered IOL positioning in all cases.

Postoperative lenticular astigmatism, calculated as the vector difference between postoperative manifest refractive astigmatism and postoperative corneal astigmatism, was evaluated in all 12 eyes (mean 0.74 ± 0.51 D). Postoperative lenticular astigmatism was 0.74 ± 0.51 D. Individual manifest refractive, corneal, and lenticular astigmatism values are provided in Supplementary Table 1.

Postoperative complications occurred in 2 eyes (16.7%). Transient anterior chamber hyphema was observed in one eye and resolved spontaneously within one week. One eye required suture revision at 2 weeks postoperatively because of IOL decentration caused by insufficient suture tension. After trimming and re-flanging the loosened suture, stable IOL centration was restored. Consequently, all IOLs were well-centered at the final follow-up. No cases of endophthalmitis, retinal detachment, chronic inflammation, elevated intraocular pressure, hypotony, or cystoid macular edema were observed during follow-up. In eyes with compromised visualization (*n* = 4), the procedure was successfully completed without additional corneal procedures. The infusion-free approach was effective in maintaining anterior chamber stability, including in a vitrectomized eye (Table 1).

**Table 1:** Patient Demographics and Clinical Outcomes

Case	Indication	Comorbidity	CDVA (logMAR)		IOP (mmHg)		ECD (cells/mm²)		IOL Decentration (Pupil Center Offset)	Complications	F/U (mo)	Final IOL Centration	Infusion System
			Pre	Post	Pre	Post	Pre	Post	x/y (mm) / Total (mm)				
1	IOL dislocation	GCD, amblyopia	1.85	1.85	12	14	3233	3123	0.21 / 0.41 / 0.46	–	5	Well-centered	–
2	Aphakia	–	0.82	0.20	17	19	3115	3004	0.21 / 0.11 / 0.24	–	5	Well-centered	–
3	IOL dislocation	PBK	1.00	0.70	12	16	415	378	0.11 / 0.38 / 0.40	–	4	Well-centered	–
4	IOL dislocation	–	0.30	0.15	17	13	1605	1599	0.01 / 0.22 / 0.22	–	4	Well-centered	–
5	IOL dislocation	Vitrectomized eye, BRVO	0.52	0.40	15	14	1639	1619	0.30 / 0.20 / 0.36	–	4	Well-centered	–
6	IOL dislocation	–	0.40	0.00	12	10	1678	1548	0.02 / 0.03 / 0.04	Hyphema	3	Well-centered	–
7	IOL dislocation	–	0.30	0.00	13	14	3096	2976	0.24 / 0.24 / 0.34	–	3	Well-centered	–
8	Aphakia	–	0.10	0.10	18	13	2989	2979	0.31 / 0.23 / 0.39	–	3	Well-centered	–
9	IOL dislocation	Corneal opacity	2.30	0.49	16	18	1759	1481	0.37 / 0.21 / 0.43	–	2	Well-centered	–
10	Zonulysis	ACG	1.70	0.22	13	10	1857	1812	0.40 / 0.04 / 0.40	–	2	Well-centered	–
11	Aphakia	–	0.20	0.10	18	16	2871	3086	0.07 / 0.10 / 0.12	–	2	Well-centered	–
12	IOL dislocation	PBK	0.82	0.30	16	12	N/A	N/A	0.07 / 0.34 / 0.35	Suture revision	2	Well-centered	–
**Mean ± SD**			**0.86 ± 0.72**	**0.38 ± 0.51**	**14.9 ± 2.4**	**14.1 ± 2.8**	**2205 ± 907†**	**2146 ± 926†**	**/0.31 ± 0.13**				

CDVA = corrected distance visual acuity; IOP = intraocular pressure; ECD = endothelial cell density

GCD = granular corneal dystrophy; PBK = pseudophakic bullous keratopathy; BRVO = branch retinal vein occlusion; ACG = angle closure glaucoma; F/U = follow-up

logMAR: lower value = better vision. 0.00 = 20/20 (decimal 1.0); HM (hand motion) ≈ 2.30; FC at 50 cm ≈ 1.85

IOL Decentration: pupil center offset x / y / total magnitude (mm), measured by anterior segment OCT. Total = √(x² + y²). All values < 0.5 mm threshold for clinical significance

† ECD mean and SD calculated from 11 eyes (Case 12 excluded: specular microscopy not obtainable due to corneal pathology)

– = none / not applicable

## Discussion

Secondary IOL fixation remains a technically challenging procedure in eyes without adequate capsular support. Conventional sutured scleral fixation techniques are associated with complications such as suture erosion, knot exposure, IOL decentration, and late suture breakage, particularly with 10 − 0 polypropylene [12–14]. 

The Yamane flanged intrascleral haptic fixation technique has emerged as one of the most widely adopted sutureless approaches, offering reliable visual recovery and avoiding suture-related long-term complications [4, 5]. However, Yamane-based fixation relies on externalization of the haptic tips of a three-piece IOL through fine scleral tunnels, which requires precise intraocular docking and carries a risk of haptic kinking or breakage. Its two-point, single-axis fixation geometry also introduces a degree of residual IOL tilt in some eyes [5, 10]. A systematic review and meta-analysis comparing Yamane and sutured scleral fixation found similar visual outcomes between the two approaches, but noted persistent variability in IOL tilt measurements, with reported tilt values of approximately 8 degrees across techniques when measured by AS-OCT [15]. 

To address the tilting risk inherent to two-point fixation, Canabrava et al. introduced the four-point flanged technique using a four closed-loop haptic foldable IOL (Artis PE, Cristalens), in which 6 − 0 or 5 − 0 polypropylene sutures are passed through the eyelets and thermally flanged at four scleral sites. Five-year data from the original Canabrava series demonstrated excellent long-term IOL stability with this knotless design [3, 6]. A prospective randomized study comparing four-point scleral fixation with the Yamane technique found comparable CDVA at one year, but significantly better refractive predictability (total refractive error − 0.06 ± 0.71 D versus 0.83 ± 0.70 D) and a similar safety profile in the four-point group, with only 0.9% endothelial cell density (ECD) loss compared with 3.5% in the Yamane group, a difference that was not statistically significant [9]. These results suggest that four-point fixation may confer favorable refractive outcomes as previously reported and, potentially, reduced endothelial stress compared with two-point Yamane fixation. Although the stable perioperative ECD values suggest preserved corneal endothelial health, the present case series was limited by its small sample size and the absence of a direct comparator group. Further comparative studies are needed to validate these findings.

The key distinction of the present trans-ocular threading technique from the original Canabrava four-point method lies in the elimination of intraocular suture manipulation. In conventional four-point flanged fixation, the surgeon must pass a needle through each closed-loop haptic while the haptic is positioned within the anterior chamber, which demands precise instrument control and adequate visualization. Our modification instead employs a full trans-ocular needle pass from sclera to contralateral corneal incision; the suture is engaged extraocularly in the needle lumen and retracted through the scleral entry, allowing each haptic to be threaded without the surgeon introducing any suture material inside the eye. This approach reduces the potential for inadvertent endothelial contact, iris trauma, or instrument-related complications.

The Carlevale IOL (FIL-SSF; SOLEKO) is another alternative for sutureless scleral fixation, employing a single-piece IOL with T-shaped haptic anchors that secure within scleral tunnels without sutures. Several studies have reported favorable outcomes with the Carlevale system, including good centration and a low complication profile [16, 17]. In contrast to the Artis PE used in our technique, the Carlevale IOL relies on passive mechanical anchoring of the T-shaped haptic flanges within scleral tunnels and requires pars plana vitrectomy in most published series. The Artis PE used in the present technique provides active suture-mediated fixation through four closed-loop haptics, enabling adjustable tension during surgery and the possibility of postoperative suture revision through a small conjunctival incision without IOL exchange—as demonstrated in one case in this series. Either platform may be appropriate depending on surgeon familiarity and local availability; the choice between them should be guided by the individual patient’s anatomy, prior surgical history, and surgeon experience.

Our technique appears potentially useful in cases with reduced anterior segment visualization, including eyes with pseudophakic bullous keratopathy, corneal opacity, or granular corneal dystrophy. In the present series, four eyes (33.3%) had concurrent corneal comorbidities that compromised intraoperative visualization; all were successfully completed without additional corneal procedures. This infusion-free approach eliminates additional instrumentation and surgical complexity, and is potentially useful in vitrectomized eyes where reduced posterior pressure could otherwise destabilize the chamber. Although the anterior chamber was maintained solely with viscoelastic in the present series, this approach should be interpreted cautiously given the limited sample size.

Regarding aphakic indications, the trans-ocular threading technique was used in three aphakic eyes in this series where the Yamane approach was not chosen. While the Yamane technique is a well-established and effective option for aphakia, our technique was preferred in these cases because of concurrent poor visualization (*n* = 2) or the absence of an infusion system, which made intraocular docking of a three-piece IOL haptic less predictable.

Despite these advantages, precise control of suture tension remains essential for optimal IOL positioning, and a learning curve is expected, similar to other flanged fixation techniques. In one of our early cases, slight postoperative IOL decentration was noted due to insufficient suture tension and was successfully corrected by adjusting the existing polypropylene flange through a small conjunctival incision.

Several limitations should be acknowledged in this study. First, this retrospective case series lacked a direct control group. While prior literature suggests potential advantages of four-point fixation over the Yamane technique and other scleral fixation methods, no direct comparison was performed within our cohort, and such claims should be interpreted cautiously pending comparative studies. Second, the mean follow-up was relatively short (3.2 ± 1.1 months), which may not have captured late complications such as flange erosion, progressive IOL decentration, suture loosening, IOP fluctuation, and chronic inflammation. Longer follow-up is needed to confirm long-term stability. Third, formal IOL tilt measurements were not performed. In addition, reliable preoperative refractive measurements were unavailable in several eyes because of aphakia, complete IOL dislocation, or media opacity; therefore, postoperative changes in astigmatism could not be assessed. As an initial case series describing a novel technique, this study should be followed by larger, longer-term studies to further validate its outcomes.

## Conclusion

The trans-ocular threading technique enables four-point flanged scleral fixation with minimal intraocular manipulation and stable anterior chamber conditions without the need for an infusion system. By converting intraocular suture handling to an extraocular procedure, this technique provides an alternative approach to a technically demanding step of conventional four-point flanged fixation and may expand access to secondary IOL implantation in cases with compromised visualization, iris damage, or other challenging anterior segment conditions. In this early case series, the technique achieved consistent IOL centration without vision-threatening complications during the short follow-up period. Corneal endothelial cell density was preserved at early follow-up, consistent with the atraumatic nature of the approach. Further validation in larger, prospective, and comparative studies with extended follow-up is required to establish long-term efficacy, suture durability, and the technique’s position relative to the Yamane and other established fixation methods.

## Supplementary Information

Below is the link to the electronic supplementary material.

Supplementary Material 1: Supplementary Table 1. Postoperative Corneal, Refractive, and Lenticular Astigmatism in Individual Eyes

Supplementary Material 2: Supplementary Video 1

## Data Availability

The data that support the findings of this study are available from the corresponding author upon reasonable request.

## References

[1] Agarwal A, Kumar DA, Jacob S, et al. Fibrin glue-assisted sutureless posterior chamber intraocular lens implantation in eyes with deficient posterior capsules. J Cataract Refract Surg. 2008;34:1433–8. 10.1016/j.jcrs.2008.04.040 18721701

[2] Gabor SG, Pavlidis MM. Sutureless intrascleral posterior chamber intraocular lens fixation. J Cataract Refract Surg. 2007;33(11):1851–4. 10.1016/j.jcrs.2007.07.013 17964387

[3] Canabrava S, Bernardino L, Batisteli T, et al. Double-flanged-haptic and capsular tension ring or segment for sutureless fixation in zonular instability. Int Ophthalmol. 2018;38:2653–62. 10.1007/s10792-017-0746-5 29067533

[4] Yamane S, Sato S, Maruyama-Inoue M, et al. Flanged intrascleral intraocular lens fixation with double-needle technique. Ophthalmology. 2017;124:1136–42. 10.1016/j.ophtha.2017.03.036 28457613

[5] Yamane S, Inoue M, Arakawa A, Kadonosono K. Sutureless 27-gauge needle-guided intrascleral intraocular lens implantation with lamellar scleral dissection. Ophthalmology. 2014;121(1):61–6. 10.1016/j.ophtha.2013.08.043 24148655

[6] Canabrava S, Carvalho MS. Double-flanged polypropylene technique: 5-year results. J Cataract Refract Surg. 2023;49(6):565–70. 10.1097/j.jcrs.0000000000001154 36745851

[7] Canabrava S, Andrade N, Henriques PR. Scleral fixation of a 4-eyelet foldable intraocular lens in patients with aphakia using a 4-flanged technique. J Cataract Refract Surg. 2021;47(2):265–9. 10.1097/j.jcrs.0000000000000310 32675655

[8] Canabrava S, Carvalho MS, Mahler O, et al. Modified Canabrava technique with a 4-closed loop intraocular lens injected into the anterior chamber. JCRS Online Case Rep. 2024;12:e00140.

[9] Błagun N, Krix-Jachym K, Rękas M. Comparison of safety and efficacy of four-point scleral intraocular lens fixation and the Yamane technique. Ophthalmol Ther. 2024;13(7):1955–66. 10.1007/s40123-024-00962-7 PMC11178701 38771460

[10] Kim J, Lee PY, Park MS, Cho BJ, Kwon S. Comparison of outcomes between modified double-flanged sutureless scleral fixation and conventional sutured scleral fixation. Sci Rep. 2024;14(1):16111. 10.1038/s41598-024-66762-y PMC11245608 38997328

[11] Mehta R, Aref AA. Intraocular Lens Implantation In The Ciliary Sulcus: Challenges And Risks. Clin Ophthalmol. 2019;13:2317–23. 10.2147/OPTH.S205148 PMC6885568 31819356

[12] Vote BJ, Tranos P, Bunce C, Charteris DG. Long-term outcome of combined pars plana vitrectomy and scleral-fixated intraocular lens implantation. Am J Ophthalmol. 2006;141(2):308–12. 10.1016/j.ajo.2005.09.012 16458685

[13] Wagoner MD, Cox TA, Ariyasu RG, Jacobs DS, Karp CL. Intraocular lens implantation in the absence of capsular support: a report by the American Academy of Ophthalmology. Ophthalmology. 2003;110(4):840–59. 10.1016/s0161-6420(02)02000-6 12689913

[14] McAllister AS, Hirst LW. Visual outcomes and complications of scleral-fixated posterior chamber intraocular lenses. J Cataract Refract Surg. 2011;37(7):1263–9. 10.1016/j.jcrs.2011.02.023 21700103

[15] Pham D, Thng S, Yuen YS, et al. Clinical outcomes in scleral fixation secondary intraocular lens with Yamane versus suture techniques: a systematic review and meta-analysis. J Clin Med. 2024;13(11):3071. 10.3390/jcm13113071 PMC11173341 38892783

[16] Barca F, Caporossi T, de Angelis L, et al. Trans-scleral plugs fixated IOL: a new paradigm for sutureless scleral fixation. J Cataract Refract Surg. 2020;46:716–20. 10.1097/j.jcrs.0000000000000135 32358266

[17] Implantation of Sutureless Scleral-Fixated Carlevale Intraocular. Lens (IOL) in Patients with Insufficient Capsular Bag Support: A Retrospective Analysis of 100 Cases at a Single Center. J Clin Med. 2025;14(11):4378. 10.3390/jcm14124378 PMC12194773 40566120

