# Supplementary material for: Early clinical outcomes of the trans-ocular threading technique for four-point flanged scleral fixation of intraocular lenses: a case series
Source: BMC Ophthalmol. 2026 Aug 7;26:723. doi: 10.1186/s12886-026-05197-7 (PMC13732985; doi:10.1186/s12886-026-05197-7)
Supplement: Supplementary file 1 [file 12886_2026_5197_MOESM1_ESM.docx]

Supplementary Table 1. Postoperative Corneal, Refractive, and Lenticular Astigmatism in Individual Eyes

| **Case** | **Indication** | **Postop Corneal Astigmatism (KRT)** | | **Postop Refractive Astigmatism** | | **Lenticular Astigmatism** | | **Notes** |
| --- | --- | --- | --- | --- | --- | --- | --- | --- |
|  |  | **Cyl (D)** | **Axis** | **Cyl (D)** | **Axis** | **D** | **Axis** |  |
| **1** | IOL disloc. | -0.75 | 70° | -1.00 | 50° | 0.64 | 116° |  |
| **2** | Aphakia | -3.50 | 170° | -2.50 | 9° | 2.17 | 147° |  |
| **3** | IOL disloc. | -0.50 | 124° | -1.00 | 121° | 0.51 | 28° |  |
| **4** | IOL disloc. | -1.00 | 14° | -0.50 | 29° | 0.62 | 2° |  |
| **5** | IOL disloc. | -1.00 | 100° | -1.50 | 102° | 0.51 | 16° |  |
| **6** | IOL disloc. | -0.25 | 52° | -0.75 | 51° | 0.50 | 141° |  |
| **7** | IOL disloc. | 0.00 | -- | -0.75 | 84° | 0.75 | 174° |  |
| **8** | Aphakia | -0.25 | 43° | -0.50 | 86° | 0.54 | 10° |  |
| **9** | IOL disloc. | -1.00 | 119° | -0.50 | 70° | 1.18 | 131° |  |
| **10** | Zonulysis | -0.75 | 40° | -0.50 | 56° | 0.42 | 20° |  |
| **11** | Aphakia | -0.50 | 118° | -0.75 | 117° | 0.25 | 25° |  |
| **12** | IOL disloc. | -1.75 | 52° | -1.50 | 65° | 0.77 | 23° |  |
